# A Detection and Tracking Method Based on Heterogeneous Multi-Sensor Fusion for Unmanned Mining Trucks

**DOI:** 10.3390/s22165989

**Published:** 2022-08-11

**Authors:** Haitao Liu, Wenbo Pan, Yunqing Hu, Cheng Li, Xiwen Yuan, Teng Long

**Affiliations:** CRRC Zhuzhou Institute Co., Ltd., Zhuzhou 412001, China

**Keywords:** unmanned mining trucks, point cloud segmentation, shape estimation, multi-source information fusion, obstacle detection

## Abstract

There exist many difficulties in environmental perception in transportation at open-pit mines, such as unpaved roads, dusty environments, and high requirements for the detection and tracking stability of small irregular obstacles. In order to solve the above problems, a new multi-target detection and tracking method is proposed based on the fusion of Lidar and millimeter-wave radar. It advances a secondary segmentation algorithm suitable for open-pit mine production scenarios to improve the detection distance and accuracy of small irregular obstacles on unpaved roads. In addition, the paper also proposes an adaptive heterogeneous multi-source fusion strategy of filtering dust, which can significantly improve the detection and tracking ability of the perception system for various targets in the dust environment by adaptively adjusting the confidence of the output target. Finally, the test results in the open-pit mine show that the method can stably detect obstacles with a size of 30–40 cm at 60 m in front of the mining truck, and effectively filter out false alarms of concentration dust, which proves the reliability of the method.

## 1. Introduction

In the open-pit mines production system, a number of mining trucks transport rocks and minerals between the power shovel and the unloading point. Due to the large blind spot and long braking distance of mining trucks, the probability of mining truck accidents is high. With the development of technology, unmanned mining trucks came into being [1]. Unmanned mining trucks improved safety and productivity while saving on labor costs, making them an integral part of the digitization of mines [2]. Environmental perception is a very important part of unmanned mining truck technology.

Scholars in the field of autonomous driving have proposed a variety of obstacle detection methods to deal with the detection of common objects on urban structured roads, such as motor vehicles, pedestrians, and bicycles [3,4,5,6,7]. Currently, the commonly used sensors for autonomous driving include Lidar, millimeter-wave radar, and a camera [8,9,10]. The camera sensor has a low cost and can obtain the shape and color of the target. Currently, it is mostly used with deep learning methods, using large-scale real-time scenes to train neural networks to achieve target size detection and type recognition [11,12,13,14]. However, the camera sensor is easily affected by the sand and dust in the environment. In recent years, the thermal imaging camera has become the trend in multi-target detection and tracking [15,16,17], which is not easily affected by dust, and has the potential to be applied to environmental perception in open-pit mines. 

At present, the industry mainly chooses Lidar and millimeter-wave radar, which are less sensitive to dust, as the main sensors for unmanned mining vehicles. The Lidar environment perception technology has high perception accuracy and a long detection distance, which is suitable for the requirements of high-level autonomous driving for sensor accuracy and stability. Therefore, based on the Lidar point cloud, the target detection algorithm has always been a hot research area. The deep learning method based on the point cloud has low detection accuracy for irregular targets lacking training samples [18,19,20]. The commonly used laser point cloud processing methods based on geometric features mainly include the Euclidean Clustering algorithm, RANSAC-based point cloud segmentation algorithm, and depth-map-based segmentation clustering algorithm [21,22,23], etc. These methods have been shown to be successful in some tasks, but the good results obtained by these methods are limited to structured road [9,24]. 

Millimeter-wave radar is not affected by conditions such as light, bad weather, and dust, with accurate speed and distance measurement performance, so it has also been widely used in the field of autonomous driving [7,8]. The target point trace directly output by the millimeter-wave radar is similar to the target after Lidar point cloud clustering. Therefore, the multi-target tracking algorithm is used to update the current position and velocity of the measurement target, and at the same time predict the position and velocity of future moment. Thereby, the position and velocity information of the target can be quickly obtained [7,25,26]. However, millimeter-wave radar also has some disadvantages. For example, the angular resolution of radar is relatively low and the clutter cannot be completely filtered out of radar measurements, leading to false detections which are hard to eliminate in the subsequent data processing [8,27]. 

At present, it is difficult for a single sensor to meet the high-precision and high-robustness requirements of the perception system of unmanned mining trucks [7,10,28]. In addition, if a single sensor fails, the safety of the unmanned mining truck cannot be guaranteed. Multi-sensor fusion methods can take advantage of all sensors to improve the robustness and redundancy of the perception system [29,30,31]. The Self-Training Multimodal Vehicle Detection Network (ST-MVDNet) is proposed to reduce the negative impact of one of the sensors being unavailable or missing on the system [32]. In [33], a novel and efficient sensor fusion system for tracking multiple moving vehicles is proposed that utilizes Lidar and Radar to improve the estimation accuracy and maximum perceived distance of target vehicles. These multi-sensor fusion methods have been shown to improve the environmental adaptability and stability of perception systems, but the results are obtained only for common obstacle detection on structured roads. Therefore, there is a lack of mature methods for dust filtration and small irregular obstacle detection on transportation roads in open-pit mines. 

The production environment of open-pit mines is complex and requires high safety. Therefore, the perception system of unmanned mining trucks needs to have the following capabilities: obtaining rapid point cloud segmentation in unpaved road environments; acquiring accurate detection of small irregular obstacles; achieving stable detection and tracking of targets in dusty environments. In order to overcome the above difficulties, the main contributions of this paper are as follows: (1) A new method for multi-target detection and tracking based on the fusion of Lidar and millimeter-wave radar is proposed to adapt to the open-pit mine environment; (2) A secondary segmentation algorithm suitable for the long-distance detection of small irregular obstacles is proposed. Firstly, the cluttered point cloud is quickly segmented into ground point cloud and non-ground point cloud based on gradient features. Then, the secondary segmentation and extraction of small obstacles misclassified as ground point clouds is realized through the feature information of single point intensity, density, gradient, etc., so that small obstacles can be detected at a longer distance; (3) The self-adaptive multi-source fusion strategy of filtering dust is adopted, which can filter the dust interference by adaptively adjusting the confidence level of the output target and the target motion characteristics, so as to reduce the false alarms and omissions caused by the open-pit mine dust; (4) Finally, the method has been applied to the actual transportation operation of the open-pit mine, and its stability has been verified under various working conditions.

The remainder of this paper is organized as follows. The heterogeneous multi-sensor fusion algorithm is proposed in Section 2. Section 3 presents the experimental results, and the proposed method is discussed in Section 4. Finally, Section 5 concludes this paper.

## 2. Method 

### 2.1. System Framework

**Figure 1:**
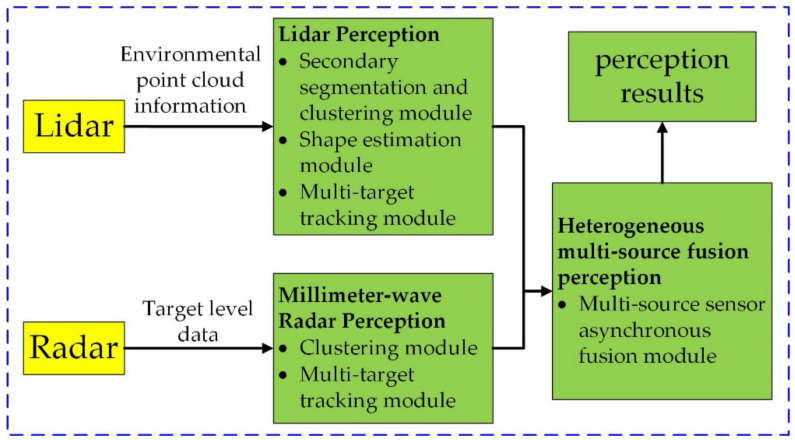
The framework of the multi-target detection and tracking method diagram.

This paper designs a multi-target detection and tracking method based on 3D Lidar and millimeter-wave radar. The system is composed of Lidar perception, millimeter-wave radar perception, and heterogeneous multi-source fusion perception. The secondary segmentation and clustering module, shape estimation module, multi-target tracking module, and multi-source sensor asynchronous fusion module cooperate with each other to achieve high real-time performance and high reliability. The perception system is shown in Figure 1.

#### 2.1.1. Lidar Perception

Innovusion’s multi-line Lidar was selected as the primary sensor. It can provide an environmental point cloud including 3D coordinates and intensity information for the perception system. The segmentation and clustering module, shape estimation module, and multi-target tracking module are used in Lidar perception to provide the fusion module with information such as target location and size.

#### 2.1.2. Millimeter-Wave Radar Perception

Millimeter-wave radar perception adopts Continental ARS408-21 mm-wave radar, which is attached to the front of the unmanned mining trucks. The radar can provide the perception system with information, such as the two-dimensional position, velocity, category, and confidence of obstacles. The results generated by the millimeter-wave radar are correlated and managed by the multi-target tracking module based on the track information, which provides reliable target data for the heterogeneous multi-source fusion perception.

#### 2.1.3. Heterogeneous Multi-Source Fusion Perception

Heterogeneous multi-source fusion perception fuses the data from millimeter-wave radar perception and Lidar perception based on the characteristics of each sensor. Due to the different sampling periods of millimeter-wave radar and Lidar, there is a time difference in synchronization. The heterogeneous multi-source fusion perception module adopts an asynchronous fusion strategy to improve the accuracy of fusion results.

### 2.2. Secondary Segmentation and Clustering Module

In order to improve the detection distance and accuracy of small irregular obstacles on unpaved roads, this paper presents a point clouds secondary segmentation algorithm adapted to the open-pit mines production scenarios. First, the disordered 3D point cloud is transformed into an ordered 2D point cloud through the point cloud depth map. Additionally, gradient features are quickly extracted using ordered 2D point clouds, thereby segmenting all point clouds into ground point clouds and non-ground point clouds according to the gradient features. Then, secondary segmentation is used to re-segment the small obstacles that were misclassified as ground points in the first segmentation, so that small obstacles can be detected at longer distances.

#### 2.2.1. Point Cloud Depth Map Construction

In the preprocessing stage, the acquired disordered point cloud data is structured. This paper converts point cloud data into depth map data according to the angular resolution of the point cloud in both horizontal and vertical directions. The ranks of the depth map are determined by the following Equations (1) and (2), respectively:

(1) $$r=\frac{V_{AG}}{V_{R}}$$

(2) $$c=\frac{H_{AG}}{H_{R}}$$

where: $V_{AG}$ is the vertical angle of view of the Lidar. $V_{R}$ is the vertical angular resolution of the Lidar. r represents the number of rows corresponding to the conversion of cloud data into a depth map. $H_{AG}$ is the horizontal field of view of the Lidar. $H_{R}$ is the horizontal angular resolution of the Lidar. c is the number of columns corresponding to the conversion of point cloud data into a depth map. In addition, down-sampling can be performed by increasing both $V_{R}$ and $H_{R}$ values.

#### 2.2.2. Point Cloud First Segmentation

**Figure 2:**
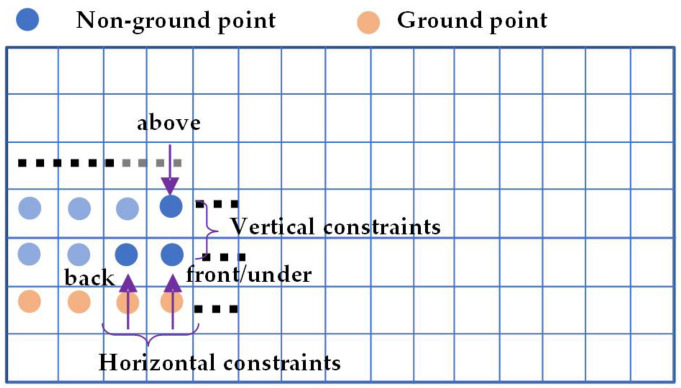
Point cloud constraint diagram.

Based on the structured point cloud depth map, the point cloud data is searched, and in the search process of which, segmentation and clustering are performed according to the feature constraints of the horizontal adjacent points and the vertical adjacent points. Among them, the horizontally adjacent points refer to the points located in the same row and the previous column in the depth map data; the vertical adjacent points refer to the points located in the same column and the next row in the depth map data. The feature constraint of the horizontally adjacent points is the Euclidean clustering or Manhattan distance between two adjacent points; the vertical adjacent point is constrained to the vertical gradient feature, and the vertical gradient is determined by Equation (3), as shown in Figure 2.

(3) $$\alpha =\arctan\left(\frac{z_{p}-z_{m}}{l_{p}-l_{m}}\right)$$

**Figure 3:**
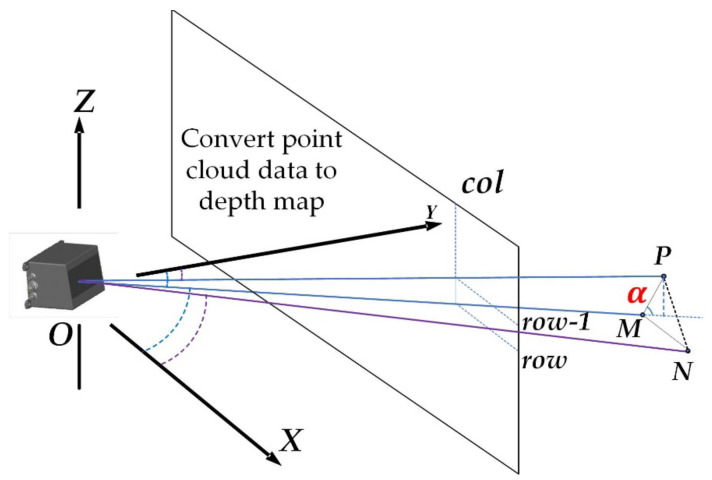
Point cloud vertical gradient feature diagram.

For example, in Figure 3, point P and point M are two points in the same column and adjacent row. 

In the equation: $z_{p}$ and $z_{m}$ respectively represent the Z-axis direction coordinate of the two points in the coordinate system of Figure 3. $l_{p}$ and $l_{m}$ respectively represent the distances from the points P and M to the coordinate origin O in the coordinate system of Figure 3. $l_{p}$ and $l_{m}$ are obtained from Equations (4) and (5), respectively:

(4) $$l_{p}=\sqrt{x_{p}^{2}+y_{p}^{2}}$$

In the equation: $x_{p}$ and $y_{p}$ are the X-axis coordinate and Y-axis coordinate of point P, respectively.

(5) $$l_{m}=\sqrt{x_{m}^{2}+y_{m}^{2}}$$

In the equation: $x_{m}$ and $y_{m}$ are the X-axis coordinate and Y-axis coordinate of point M, respectively.

As shown in Figure 2, the distance between the point (point pointed to by the front pointer) and the adjacent points (point pointed to by the back pointer) in the same row and previous column is used to determine whether the horizontal constraint condition is satisfied. This algorithm calculates the vertical gradient feature *α* between the current point (the point pointed to by the under pointer) and its vertical adjacent point (the point pointed to by the above pointer) according to Equation (3). By comparing the vertical gradient feature *α* with the threshold *α*_0_, if it is greater than the threshold *α*_0_, it is determined that the point and the vertical adjacent point are non-ground points and belong to the same obstacle. If it is less than the threshold *α*_0_, the point and the vertical adjacent points are regarded as near-ground points.

#### 2.2.3. Secondary Segmentation 

The secondary segmentation is to re-segment the small obstacles that were wrongly classified as ground points in the first segmentation, so that small obstacles can be detected at a longer distance. First, the region of interest is re-segmented with different thresholds based on the near-ground points obtained from the first segmentation. For near-ground points, by setting the gradient thresholds of point clouds in different regions, the point clouds of near-ground objects can be extracted more precisely, thereby ensuring that small objects can be segmented to obtain more point clouds. Second, an obstacle point cloud is extracted based on single point intensity and neighborhood gradient. In general, the point cloud intensity of obstacles will be significantly higher than that of the ground point cloud. In the ground point cloud after the first segmentation and re-segmentation of the region of interest, the point cloud with prominent intensity is used as the seed point. After the seed point is found, the gradient characteristics of the points in its neighborhood are searched. If it meets the threshold requirements, the point cloud in this area is divided into non-ground point clouds. Third, small obstacles are extracted based on the density characteristics of the point cloud.

**Figure 4:**
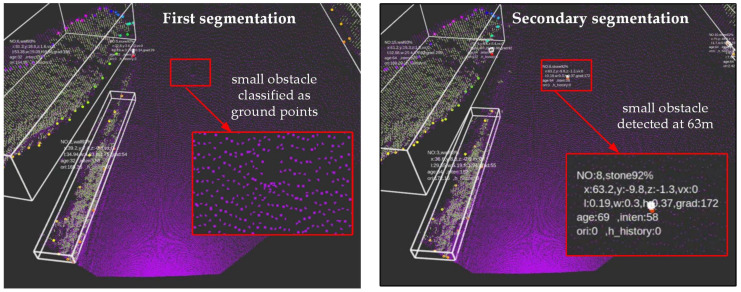
The detection effect of first segmentation and secondary segmentation. (**a**) Detection effect based on first segmentation. (**b**) Detection effect based on secondary segmentation.

Figure 4 shows the detection results based on first segmentation and secondary segmentation, respectively. It can be seen that the perception system based on secondary segmentation can detect small obstacles at a distance of 63 m.

### 2.3. Clustering

Both DBSCAN and Optics are very useful clustering algorithms. Among them, the Optics algorithm is not sensitive to parameter settings, and is more stable for multi-objective clustering in complex environments [34]. However, the targets in the open-pit mine scene are mainly vehicles, retaining walls and stones, etc., and there is no situation where multiple vehicles are very close to each other. Compared with the Optics algorithm, the DBSCAN algorithm requires less computation and can better meet the real-time requirements [35]. Therefore, the DBSCAN algorithm is selected to cluster segmented non-ground point clouds and the results of the millimeter-wave radar. The algorithm mainly uses two parameters: radius *eps* and density threshold *Minpts*. A combination of the characteristics and resolution of the sensor and the installation position determine the *eps* and *Minpts* parameters in different areas in front of and on the side of the mining truck. The *eps* and *Minpts* parameters of different areas in the front and side of the mining truck are determined by combining the characteristics and resolution of the sensor and the installation position. Mahalanobis distance is used instead of Euclidean distance when calculating point-to-point distances. The spatial coordinates, speed, and strength of obstacles are selected as Mahalanobis distance parameters. The fundamental purpose of the clustering algorithm is to divide the scattered target points into several independent point sets, which are regarded as obstacles.

### 2.4. Shape Estimation Module

**Figure 5:**
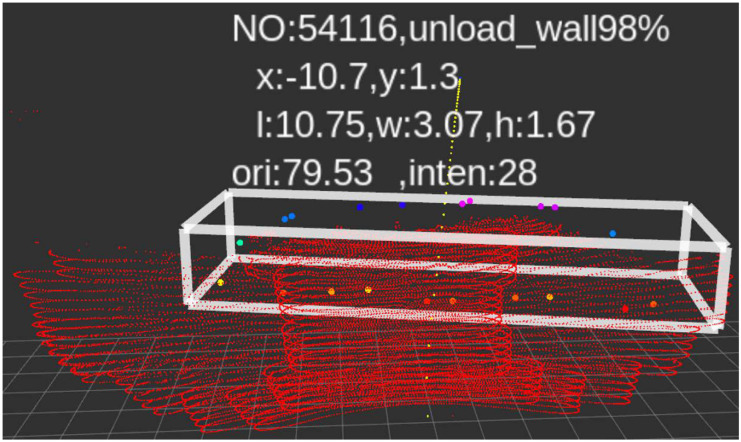
Angle estimation of the retaining wall.

For the point cloud of the target obtained after segmentation and clustering, it is also necessary to estimate its corresponding orientation, position, and other information. For example, in the process of unloading, in order to ensure that all the goods can be unloaded outside the retaining wall, the left and right rear wheels need to be close to the retaining wall when unloading. Therefore, it is necessary to get the angle of the retaining wall in advance. The commonly used shape estimation, such as point-to-edge squared error minimization, is not accurate and has low real-time performance. Shape estimation based on a convex hull model relies on convex hull points and the accuracy is also not high, which makes it difficult to meet the real-time performance of the algorithm in the opencast mine scene. Therefore, the shape fitting algorithm that maximizes the point-to-edge closeness is adopted in this paper. The shape of the wall point cloud obtained is based on the assumption of L-Shape, while the point cloud is mainly gathered at the two edges and intersections of L-Shape. The above divides the point cloud into regions according to the distribution characteristics of the point cloud, and calculates the sum of the distances d from all points to the sides of the current rectangular frame, the area a of the current rectangular frame, and the difference n between the number of point clouds in the area with the most and the least point cloud distribution. The objective function f is composed of several parameters. The expression is shown in (6). By traversing all possible facing angles of the obstacle to obtain the angle and rectangular box corresponding to the maximum value of the objective function, the facing angle and rectangle of the target can be determined. The schematic diagram of the retaining wall shape estimation is shown in Figure 5.

(6) $$f=\frac{n^{2}}{a\times d}$$

### 2.5. Multi-Target Tracking Module

In this paper, multi-target tracking is used to process the target information after the laser radar point cloud clustering and the target information directly provided by the millimeter-wave radar. Data association is carried out according to the relationship between the predicted and measured space poses, and finally the current state and track of the target are updated according to the association result, and the trajectory state of the target is managed uniformly.

#### 2.5.1. Data Association

In the process of multi-target tracking, a single predicted value will fall into the intersection area of multiple correlation gates, or multiple predicted values will fall into the correlation gate of a single target. Considering the above situation, it is necessary to establish the relationship between the radar measurement data at a certain moment and the measurement data at other moments to determine whether these measurement data come from the same target. Therefore, a deformation algorithm of the nearest neighbor data association algorithm is proposed based on the track information.

**Figure 6:**
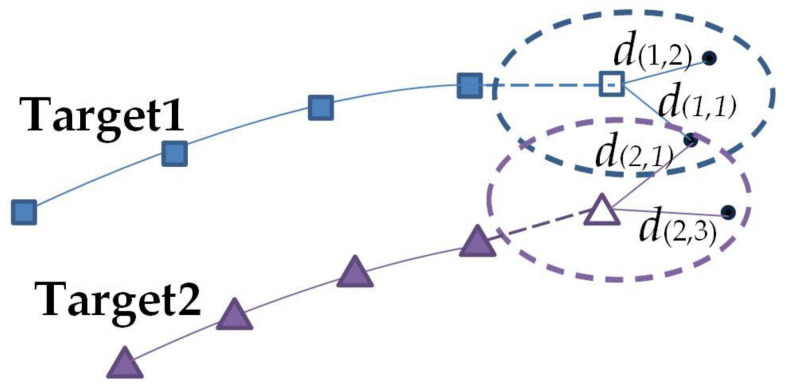
Nearest neighbor association diagram.

The algorithm analyzes the data of the previous frame of each target to obtain the predicted value of the current frame of the corresponding target, so as to obtain all the target measurement values of the current frame of the radar. Meanwhile, a correlation gate is established according to the relationship between the target predicted value and the measured value, and then the correlation distance between each predicted value and the measured value is calculated. The association distance matrix formed is shown in Equation (7). Finally, the correlation distance in each target correlation gate is traversed to find the measurement value corresponding to the minimum correlation distance to achieve data correlation. By analogy, the measurement values corresponding to the current frame of all targets can be obtained. The schematic diagram of the algorithm is shown in Figure 6.

The solid squares and triangles represent the position of the target at historical moments, and the hollow squares and hollow triangles represent the predicted values of targets 1 and 2, and the solid circles represent the measured values of the current frame of the radar. The dotted box represents the correlation gate established between the predicted value and the measured value according to the target. $d_{\left(1,1\right)}$ represents the correlation distance between target 1 and measurement 1.

The correlation distance matrix constructed by each predicted value and the measured value is:

(7) $$A=\left[\begin{array}{ccccc} d_{\left(1,1\right)} & d_{\left(1,2\right)} & \cdots & d_{\left(1,j-1\right)} & d_{\left(1,j\right)}\\ d_{\left(2,1\right)} & d_{\left(2,2\right)} & \cdots & d_{\left(2,j-1\right)} & d_{\left(2,j\right)}\\ \vdots & \vdots & \ddots & \vdots & \vdots \\ d_{\left(i-1,1\right)} & d_{\left(i-1,2\right)} & \cdots & d_{\left(i-1,j-1\right)} & d_{\left(i-1,j\right)}\\ d_{\left(i,1\right)} & d_{\left(i,2\right)} & \cdots & d_{\left(i,j-1\right)} & d_{\left(i,j\right)} \end{array}\right]$$

In the equation: $d_{\left(p,q\right)}$ represents the correlation distance between the *p*-th predicted value of the previous frame and the *q*-th measured value of the current frame, where *p*, *q* are the target lower label number. Assuming that there are *i* targets and *j* measurements in the current frame, target 1 first traverses the measurements in the correlation gate. As shown in the figure above, there are only $d_{\left(1,1\right)}$, $d_{\left(1,2\right)}$ in the correlation gate and $d_{\left(1,1\right)}>d_{\left(1,2\right)}$, and then target 1 is correlated with measurement 2. The other targets and the remaining measurement values correlate in the same way as above.

This algorithm defines the Mahalanobis distance *M_d_*, as shown in Equation (8), where *d_x_* and *d_y_* are the distance between the predicted value and the measured value in *x* and *y* directions of the vehicle coordinate system, respectively. *S_x_* and *S_y_* are the variance of the distance between the target value and the measured value in *x*, *y* directions, respectively. Then, it further defines the correlation distance between the target value and the measured value as *d*, as shown in Equation (9).

(8) $$M_{d}=\sqrt{\frac{d_{x}{}^{2}}{S_{x}}+\frac{d_{y}{}^{2}}{S_{y}}}$$

(9) $$d=\frac{M_{d}}{\log_{e}\left(t_{l}+2\right)}$$

In the equation: $M_{d}$ is the Mahalanobis distance defined above, $t_{l}$ indicates the track length of the current target, and *e* is the natural logarithm.

#### 2.5.2. Track Management

In the process of multi-objective association, the calculation of the association distance involves the current track length of the target, so the track management is adopted to realize the track generation, track retention, and track deletion in the process of multi-target tracking. Among them, the track management of the target can be represented by four track states, namely the newly generated (header), keep (keep), to-be-deleted (shade), and delete (leave) states.

If there is a new measurement value that is not associated with the track, the measurement value is used as the new track header, and the track status is the header. If the target track status is associated with measurement for several consecutive frames, its track status changes to keep, and the track will continue to be maintained. If the target track state has no measurement association at the next moment, its track state changes to shade, and the track state changes to waiting to be deleted. When the target track in the shade state has no associated measurement for several consecutive frames, its track state changes to leave, and the track is deleted.

### 2.6. Multi-Source Sensor Asynchronous Fusion Module

In order to improve the accuracy of target detection results and overcome the difficulties of heterogeneous sensor fusion, in this paper, a multi-source sensor asynchronous fusion strategy is designed to improve target detection and tracking ability on unpaved roads.

#### 2.6.1. Time-Space Synchronization of Multi-Source Sensors 

In this paper, soft synchronization is used to realize the time synchronization between multi-source sensors. The sensor data are acquired in real time through a Robot Operating System (ROS), and a uniform time is provided for the sensor data according to the clock of the computing unit.

The spatial synchronization of multi-source sensors is further divided into the spatial synchronization of Lidar and vehicle coordinate systems, and the spatial synchronization of Lidar and millimeter-wave radar. The transformation between the Lidar coordinate system relative to the vehicle coordinate system can be represented by the translation deviations Δ*x*, Δ*y*, Δ*z*, and the three rotation angle deviations *α*, *β*, *γ*, on the three coordinate axes. Therefore, this paper adopts the feature-based calibration method to calibrate the external parameters of the Lidar. First, calibrate the pitch angle deviation *α*, roll angle deviation *β*, translation parameter offset ∆*z*, then calibrate the yaw angle deviation *γ*, and finally calibrate the translation offset ∆*x*, ∆*y*, so as to complete the spatial synchronization between the Lidar and the vehicle coordinate system.

Since the target-level data obtained by the millimeter-wave radar only has the coordinate information of the *x* and *y* axes, in this paper, the multi-sensor joint calibration method is used to realize the spatial synchronization of millimeter-wave radar and Lidar. The conversion relationship of the sensor coordinate system is shown in Equation (10):

(10) $$\left\{\begin{array}{c} X_{L}=X_{R}\cos\left(\delta_{\gamma }\right)+Y_{R}\sin\left(\delta_{\gamma }\right)+\delta_{X}\\ Y_{L}=-X_{R}\sin\left(\delta_{\gamma }\right)+Y_{R}\cos\left(\delta_{\gamma }\right)-\delta_{Y} \end{array}\right.$$

In the equation: *X_L_*, *Y_L_* respectively represent the coordinate values of the *X* and *Y* directions in the Lidar coordinate system, and $X_{R}$, $Y_{R}$ respectively represent coordinate value of the *X* and *Y* directions in the millimeter-wave radar coordinate system. $\delta_{X}$, $\delta_{Y}$ represent the translation offsets on the *X* and *Y* directions , and $\delta_{\gamma }$ represents the offset of heading angle.

#### 2.6.2. Target State Fusion 

In order to solve the problems of false positives and false negatives caused by dust in the open-pit mine environment, this paper proposes an adaptive dust filtering fusion strategy. In the case of non-dust, the fusion algorithm uses fuzzy logic judgment to output the fused target according to the perception results, matching the results and tracking stability results of Lidar and millimeter-wave radar. For example, when a target is detected by both Lidar and Radar at the same time, the output confidence of the target will be improved with the increase in the number of detection frames and matching frames. When the confidence of the target reaches the threshold, the perception module will output the target to the decision module. The output confidence threshold of the object comprehensively considers the size, category, distance, and other information of the object. 

Lidar point clouds reflected from dust are sparser and weaker than common targets such as vehicles, retaining walls, and rocks. In addition, Lidar has difficulty in detecting targets behind dust, while millimeter-wave radar can detect obstacles behind dust. When a high-concentration dust scene is detected according to the features of the laser point cloud, the fusion algorithm will adaptively adjust the output confidence of the obstacles of the Lidar and millimeter-wave radar. For example, when the Lidar determines that there is a high concentration of dust ahead that blocks the field of view, the fusion algorithm uses the characteristics of millimeter-wave radar to penetrate dust perception to adaptively improve the confidence of the millimeter-wave radar to detect obstacles after dust. Therefore, the confidence of the target behind the dust can increase rapidly with the increase in the number of radar detection frames, thus reaching the threshold for outputting the target to the decision module. The adaptability and robustness of the multi-source sensor fusion algorithm in the dusty environment of open-pit mines are improved. 

Algorithm 1 provides the pseudocode of the fusion algorithm for dust scenes. The calculation methods of the influencing factor values of size, speed, tracking result, source, and matching result are given in Algorithm 1. If the calculated influence factor value is greater than 1, the maximum value is 1. The confidence of the final object is the sum of each influence factor value multiplied by the corresponding influence coefficient. The influence coefficient values will be adjusted when the detection field of view is blocked by dense dust. When the confidence of the object is greater than the set threshold, it will be output by the fusion algorithm.
**Algorithm 1** The fusion algorithm for dust scenes.**Input:** Lidar sensing results, Radar sensing results, matching results, tracking results, dust warning information.**Output:** Fusion perception results.# Calculate the influence factor value of each item. 1: length influence factor value = obj_length/length_threshold 2: width influence factor value = obj_width/width_threshold 3: height influence factor value = obj_height/height_threshold 4: velocity influence factor value = obj_vel/vel_thresold 5: track influence factor value = obj_ track_frames ^ 1.5/maximum track length 6: source of object influence factor value: only Lidar = 0, only Radar = 1, Lidar and Radar = 2 7: match influence factor value = obj_match_frames ^ 2/maximum track length# Set the default value of the influence coefficient corresponding to each influence factor. 8: Default: coeff(length) = coeff(width) = 0.3, coeff(height) = 0.5, coeff(velocity) = 0.5,   coeff(track) = 1.0, coeff(source) = 0, coeff(match) = 1.0# Determine if there is a high concentration of dust ahead blocking the view.# Calculate the confidence of object. 9: if high concentration dust == true 10:  then 11:   coeff(source) = 1.0, coeff(velocity) = 1.0, coeff(match) = 0, coeff(track) = 1.5,      coeff(height) = 0 12:    obj_confidence = Σ influence factor * coeff(influence factor) 13:  if obj_confidence > confidence _threshold 14:    then 15:      output obj 16:  end if 17: else 18:  obj_confidence = Σ influence factor * coeff(influence factor)* 19:  if obj_confidence > confidence _threshold 20:  then 21:     output obj 22:  end if 23: end if

The traditional nearest neighbor matching algorithm only considers the distance between points, which leads to the failure of the association of large-sized obstacles perceived by Lidar and millimeter-wave radar when the space-time synchronization error is large. Therefore, this paper uses Mahalanobis distance instead of Euclidean distance. The parameters such as obstacle coordinates, length, width, and heading angle, are used to calculate the Mahalanobis distance and set the variance of the corresponding parameters. Compared with the traditional nearest neighbor algorithm, the accuracy of obstacle association is improved. 

In terms of target attribute recognition, the Lidar performs a fuzzy comparison with the corresponding attributes of the classified target according to the detected target size, speed, average intensity, and other information, and initially obtains the type information of the target. In the fusion module, the detection results and matching relationship of Lidar and millimeter-wave radar are combined to confirm the target type.

## 3. Experiments and Analysis

**Figure 7:**
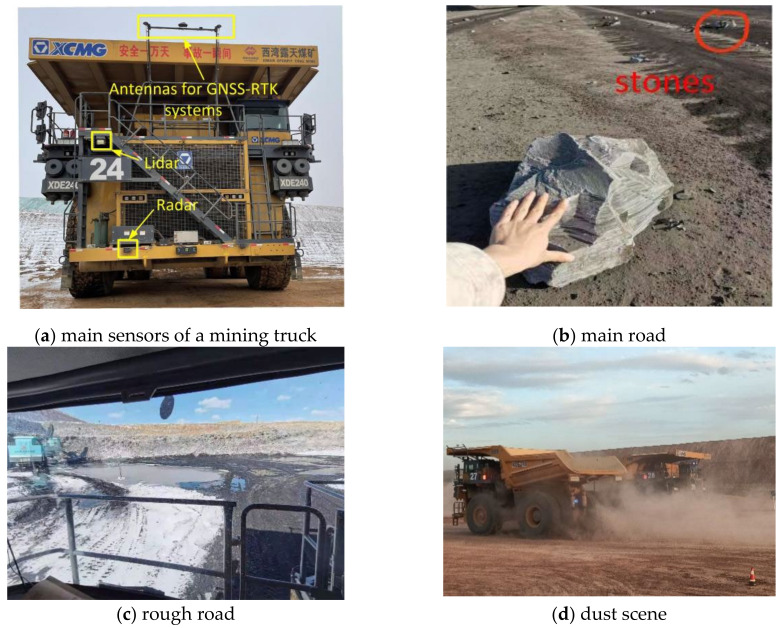
Driving environment of unmanned mining trucks.(**a**) The photo of the mine truck with Lidar and millimeter-wave radar; (**b**) The main road littered with stones; (**c**) The rough road in the loading area; (**d**) The dust scene in the unloading area.

In this paper, based on the unmanned operation environment of mining trucks, the functions of each module of the proposed multi-source sensor fusion perception system are experimentally verified. The road operation environment is shown in Figure 7. The mining truck is tested on the open-pit mine road. The test scenarios include main loading, carrying, and unloading scenarios. The system uses Lidar and millimeter-wave radar to collect raw data. Multi-target detection and tracking algorithms are used to process the data and analyze the perception results.

**Figure 8:**
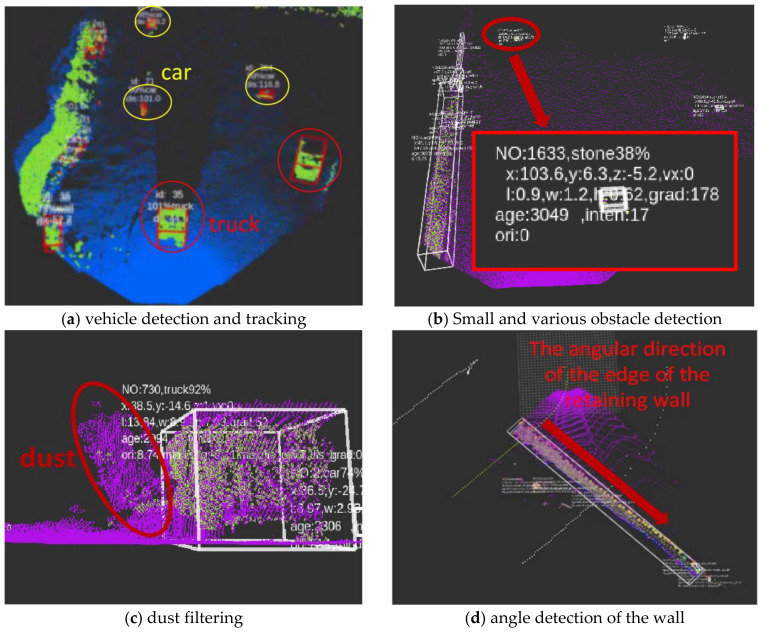
Multi-source sensor target fusion detection and tracking.

This paper verifies the effect of multi-source sensor asynchronous fusion in the actual operation scene of the open-pit mine. As the open-pit mine does not allow pedestrians to enter during operations, the targets detected in the scene are mainly vehicles, rocks, and walls. Therefore, the technical difficulties, such as moving target detection and tracking, small target detection, dust filtering, and wall angle detection, are mainly verified. Figure 8a shows the detection and tracking effect of trucks and cars. The perception system can detect trucks at 250 m, and it can stably track the ID, position, and speed of the truck at a distance of 150 m. Besides, it has a perception distance of 200 m for cars, and can track and detect stably within a range of 150 m. Figure 8b shows the detection effect of small and various obstacles. The detection distance of a 40 cm × 40 cm × 40 cm target can reach 80 m, and obstacles, such as stones and equipment with a height of more than 30 cm, can be accurately sensed within 50 m, which can avoid damage to vehicle tires. Figure 8c is a common dust-raising scene in an open-pit mine. The dust raised by the vehicle is easily mixed with the vehicle, making the volume of the vehicle detected by the Lidar larger than the actual volume, resulting in false triggering of collision and braking. By means of the insensitivity of millimeter-wave radar to dust, the point clouds returned by multiple millimeter-wave radars installed in front of the vehicle are clustered, which are fused with the Lidar perception results. By judging the difference in size and position of the millimeter-wave radar and Lidar clusters, it is determined whether the size of the obstacles reported by the Lidar in the current frame is distorted due to dust. If there is size distortion, the size and contour of the millimeter-wave radar are used for correction to filter out the dust part, thereby reducing false alarms of dust. During the unloading process, the perception system must accurately detect the angle information of the wall for the decision-making control system to ensure that the vehicle stops at the wall in the correct posture to complete the unloading work. As shown in Figure 8d, the angle of the wall can be effectively detected by the shape estimation algorithm.

## 4. Discussion

### 4.1. Results

In open-pit mine production systems, common detection objects include vehicles, retaining walls, gravel piles, and stones. Among them, the proposed heterogeneous multi-sensor system can be detected and tracked very stably due to the very large size of the vehicles and the retaining wall. However, small gravel piles and stones with different shapes are difficult to detect and track very stably, which can cause serious damage to the tires of mining trucks. Therefore, the ability of the proposed system to detect small and irregular targets is analyzed below.

**Figure 9:**
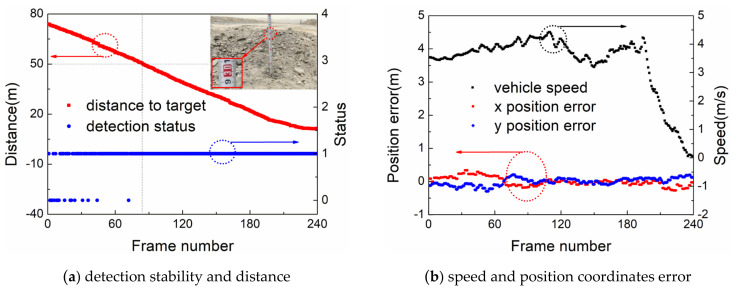
Detection and tracking effect of small gravel piles.

In Figure 9a, the red curve depicts the mine truck approaching the small gravel pile (about 30 cm high) from 73.8 m. The blue curve describes the detection effect of the proposed system for small gravel pile, “1” indicating that the small gravel pile has been detected, and “0” indicating that it has not been detected. The small gravel pile was first detected by the proposed system at a distance of 73.8 m from the mine truck. In 240 frames of data, up to 15 frames were missed. However, a total of 165 frames were detected (no frame was lost) within 53 m from the mine truck, which proves that the algorithm in this paper stably detects the small gravel piles. The coordinate system is established with the forward direction of the harvester as the positive direction of X-axis and the left side of the harvester as the positive direction of Y-axis. By combining the Universal Transverse Mercator (UTM) coordinates of the mining truck given by the GNSS TRK system and the relative position between the detected target and the vehicle body, the UTM coordinates of the small gravel pile can be obtained. Since the target is stationary, the average value of statistics is used as the true value of its UTM coordinates. The error calculation equation of position coordinates (East, North) is as follows:

(11) $$x_{i\_position\;error}=x_{i}-\frac{\sum_{i=1}^{n}x_{i}}{n}$$

(12) $$y_{i\_position\;error}=y_{i}-\frac{\sum_{i=1}^{n}y_{i}}{n}$$

If the absolute values of the difference between the maximum value, minimum value, and mean value of position coordinates are all less than 0.5 m in multiple frames, the position coordinates deviation test is considered to be qualified. The specific calibration calculation equation is:

(13) $$\sqrt{\left(\max(x_{i}\right)-\frac{\sum_{i=1}^{n}x_{i}}{n}{)}^{2}}<0.5\;\mathrm{and}\;\sqrt{\left(\min(x_{i}\right)-\frac{\sum_{i=1}^{n}x_{i}}{n}{)}^{2}}<0.5$$

(14) $$\sqrt{\left(\max(y_{i}\right)-\frac{\sum_{i=1}^{n}y_{i}}{n}{)}^{2}}<0.5\;\mathrm{and}\;\sqrt{\left(\min(y_{i}\right)-\frac{\sum_{i=1}^{n}y_{i}}{n}{)}^{2}}<0.5$$

In the equation: *i* = 1~*n* is the measured value of the test single frame sample, *n* is the total number of frames; *x_i_* and $y_{i}$ are the East and North position coordinates of the frame *i,* respectively, and the unit is m.

In Figure 9b, the black curve depicts the speed change when the mining truck approaches the small gravel pile, and the red and blue curves represent the position coordinates error of each frame of measurement value. It can be seen that the maximum error values of the x (east) and y (north) position coordinates are both less than 0.5 m during the variable speed operation of the mining truck. The accuracy of the algorithm for the position detection of small gravel piles can be proved.

**Figure 10:**
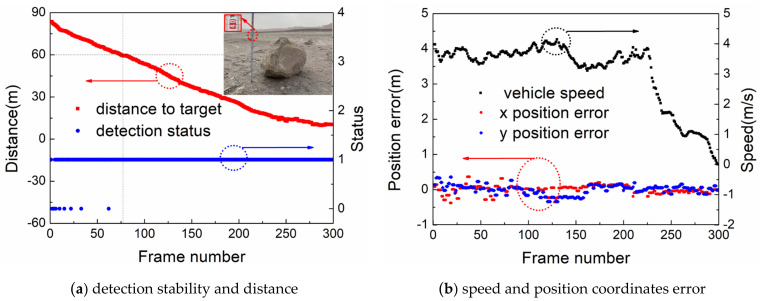
Stone detection and tracking effect.

Next, the detection and tracking ability of the proposed system for stones is verified through detection data analysis. The proposed system detected the stone (about 40 cm × 40 cm × 40 cm) for the first time at a distance of 83.7 m when the mine truck approached the stone from far to near, as shown in Figure 10a. In the 300 frames of detection data, a total of 9 frames were lost. However, when the mine truck is less than 63 m away from the stone, the stone can be successfully detected in every frame, which proves that the algorithm in this paper detects the stone stably. The maximum error values of the x (East) and y (North) position coordinates in Figure 10b are calculated by Equations (11) and (12). It can be seen that the position errors are all less than 0.45 m, which meets the requirements of position detection accuracy.

### 4.2. Time Analysis

The industrial computer selected in the experiment is based on the x86 platform, the CPU is intel6820HQ, the main frequency is 2.7 GHz, the memory capacity is 16 G, and the operating system is Ubuntu18.04. The source code of the algorithm is written in C++ language, using multi-threaded programming technology based on OpenMP library. Based on the above platform, the test is carried out in the normal working scene in the open-pit mine environment, and the average time of the perception algorithm in the operation process is taken as the final test score. The test results are as follows: In the normal loading and dumping process of mining trucks, the driving speed is about 20 km/h, the Lidar sensing algorithm takes about 90 ms, the millimeter-wave radar sensing algorithm takes about 20 ms, and the multi-source sensor fusion algorithm takes about 30 ms. It can be seen that the algorithm proposed in this paper meets the perception requirements of mining trucks in the autonomous driving environment, and has a certain redundancy in computing power. In the future, further research can be done to optimize the algorithm for parallel acceleration based on the GPU platform to meet higher real-time requirements.

## 5. Conclusion

This paper proposes a method for the detection and tracking of targets on rough and dusty roads during the driving process of mining trucks. This method uses a new secondary segmentation algorithm suitable for small object extraction on rough roads to improve the detection distance and accuracy of small irregular obstacles. It also proposes an adaptive asynchronous fusion strategy of multi-source sensors, which overcomes the problem of false positives and false negatives caused by dust to improve the detection and tracking capabilities of various targets in transportation at open-pit mines. Experiments show that the method can stably perceive vehicles within a range of 200 m, and accurately detect irregular obstacles with a size of 30–40 cm within a range of 60 m. However, this method makes it difficult to accurately distinguish between small mounds and stones. The next step will be to focus on the classification of small targets.

## Data Availability

Not applicable.

## References

[1] Yuan Y. (2013). Unmanned technology for mining trucks. Min. Equip..

[2] Widdififield L., Riggle R. (2016). The Big Picture: An Overview Approach to Surface Mining. Min. Eng..

[3] Asvadi A., Peixoto P., Nunes U. (2016). Two-stage static/dynamic environment modeling using voxel representation. Robot 2015: Second Iberian Robotics Conference.

[4] Asvadi A., Cristiano P., Peixoto P., Nunes U. (2016). 3D Lidar-Based Static and Moving Obstacle Detection in Driving Environments: An Approach Based on Voxels and Multi-Region Ground Planes. Robot. Auton. Syst..

[5] Dairi A., Harrou F., Senouci M., Sun Y. (2018). Unsupervised Obstacle Detection in Driving Environments Using Deep-Learning-Based Stereovision. Robot. Auton. Syst..

[6] Darms M.S., Rybski P.E., Baker C., Urmson C. (2009). Obstacle Detection and Tracking for the Urban Challenge. IEEE Trans. Intell. Transp. Syst..

[7] Wei H.L., Guo A.B., Dong W.B., Wang X.J., Huang G.K. (2015). Research and application of millimeter wave radar collision avoidance system for mining truck. Saf. Coal Mines.

[8] Zhou T., Yang M., Jiang K., Wong H., Yang D. (2020). MMW Radar-Based Technologies in Autonomous Driving: A Review. Sensors.

[9] Lin B. (2021). A New Challenge: Detection of Small-Scale Falling Rocks on Transportation Roads in Open-Pit Mines. Sensors.

[10] Xiao D., Shan F., Li Z., Le B.T., Liu X., Li X. (2019). A Target Detection Model Based on Improved Tiny-Yolov3 Under the Environment of Mining Truck. IEEE Access.

[11] Lu S., Luo Z., Gao F., Liu M., Chang K., Piao C. (2021). A Fast and Robust Lane Detection Method Based on Semantic Segmentation and Optical Flow Estimation. Sensors.

[12] Brabandere B.D., Neven D., Gool L.V. Semantic instance segmentation for autonomous driving. Proceedings of the 2017 IEEE Conference on Computer Vision and Pattern Recognition Workshops.

[13] Liu P., King I., Lyu M.R., Xu J. DDFlow: Learning optical flow with unlabeled data distillation. Proceedings of the Thirty-Third AAAI Conference on Artificial Intelligence.

[14] Chen L.C., Papandreou G., Kokkinos I. (2018). DeepLab: Semantic image segmentation with deep convolutional nets, atrous convolution, and fully connected CRFs. IEEE Trans. Pattern Anal. Mach. Intell..

[15] Liu Q., Lu X., He Z., Zhang C., Chen W.S. (2017). Deep convolutional neural networks for thermal infrared object tracking. Knowl. -Based Syst..

[16] Liu Q., Li X., He Z., Fan N., Yuan D., Wang H. (2020). Learning deep multi-level similarity for thermal infrared object tracking. IEEE Trans. Multimed..

[17] Liu Q., Yuan D., Fan N., Gao P., Li X., He Z. (2022). Learning dual-level deep representation for thermal infrared tracking. IEEE Trans. Multimed..

[18] Minemura K., Liau H.F., Monrroy A., Kato S. Lmnet: Real-Time Multiclass Object Detection on Cpu Using 3D Lidar. Proceedings of the 2018 3rd Asia-Pacific Conference on Intelligent Robot Systems (ACIRS).

[19] Beltran J., Guindel C., Moreno F.M., Cruzado D., Garcia F., de la Escalera A. Birdnet: A 3D Object Detection Framework from Lidar Information. Proceedings of the 2018 21st International Conference on Intelligent Transportation Systems (ITSC).

[20] Shi S., Wang X., Li H. PointRCNN: 3D Object Proposal Generation and Detection from Point Cloud. Proceedings of the IEEE/CVF Conference on Computer Vision and Pattern Recognition.

[21] Rummelhard L., Paigwar A., Negre A., Laugier C. Ground Estimation and Point Cloud Segmentation using SpatioTemporal Conditional Random Field. Proceedings of the 2017 IEEE Intelligent Vehicles Symposium (IV).

[22] Urmson C., Anhalt J., Bagnell D., Baker C., Bittner R., Clark M.N., Dolan J., Duggins D., Galatali T., Geyer C. (2008). Autonomous driving in urban environments: Boss and the urban challenge. J. Field Robot..

[23] Narksri P., Takeuchi E., Ninomiya Y., Morales Y., Akai N., Kawaguchi N. A Slope-robust Cascaded Ground Segmentation in 3D Point Cloud for Autonomous Vehicles. Proceedings of the 2018 21st International Conference on Intelligent Transportation Systems (ITSC).

[24] Chen T.T., Dai B., Wang R.L., Liu D.X. (2014). Gaussian-Process-Based Real-Time Ground Segmentation for Autonomous Land Vehicles. J. Intell. Robot. Syst..

[25] Li M., Stolz M., Feng Z., Kunert M., Henze R., Küçükay F. An adaptive 3D grid-based clustering algorithm for automotive high resolution radar sensor. Proceedings of the IEEE International Conference on Vehicular Electronics and Safety (ICVES).

[26] Scheel A., Knill C., Reuter S., Dietmayer K. Multi-sensor multi-object tracking of vehicles using high-resolution radars. Proceedings of the IEEE Intelligent Vehicles Symposium (IV).

[27] Dickmann J., Klappstein J., Hahn M., Appenrodt N., Bloecher H.L., Werber K., Sailer A. Automotive radar the key technology for autonomous driving: From detection and ranging to environmental understanding. Proceedings of the IEEE Radar Conference (RadarConf).

[28] Voronov Y., Voronov A., Makhambayev D. (2020). Current State and Development Prospects of Autonomous Haulage at Surface Mines. E3S Web Conf. EDP Sci..

[29] Haris M., Glowacz A. (2022). Navigating an Automated Driving Vehicle via the Early Fusion of Multi-Modality. Sensors.

[30] Wang Z., Wu Y., Niu Q. (2019). Multi-sensor fusion in automated driving: A survey. IEEE Access.

[31] Prakash A., Chitta K., Geiger A. Multi-modal fusion transformer for end-to-end autonomous driving. Proceedings of the IEEE/CVF Conference on Computer Vision and Pattern Recognition.

[32] Li Y.J., Park J., O’Toole M., Kitani K. Modality-Agnostic Learning for Radar-Lidar Fusion in Vehicle Detection. Proceedings of the IEEE/CVF Conference on Computer Vision and Pattern Recognition.

[33] Lee H., Chae H., Yi K. (2019). A geometric model based 2D LiDAR/radar sensor fusion for tracking surrounding vehicles. IFAC-PapersOnLine.

[34] Wagner T., Feger R., Stelzer A. Modifications of the OPTICS clustering algorithm for short-range radar tracking applications. Proceedings of the 2018 15th European Radar Conference (EuRAD).

[35] Deng D. (2020). DBSCAN clustering algorithm based on density. Proceedings of the 2020 7th International Forum on Electrical Engineering and Automation (IFEEA).

